# Study protocol for a multi-methods study: SAVOIR - evaluation of specialized outpatient palliative care (SAPV) in Germany: outcomes, interactions, regional differences

**DOI:** 10.1186/s12904-019-0398-5

**Published:** 2019-01-26

**Authors:** Antje Freytag, Markus Krause, Anna Bauer, Bianka Ditscheid, Maximiliane Jansky, Sabine Krauss, Thomas Lehmann, Ursula Marschall, Friedemann Nauck, Werner Schneider, Kathleen Stichling, Horst Christian Vollmar, Ulrich Wedding, Winfried Meißner, the SAVOIR Study group, Anna Bauer, Lia Bergmann, Bianka Ditscheid, Cornelia Eichhorn, Antje Freytag, Elke Gaser, Michaela Hach, Ulrike Hammer, Aicko Helbig, Beata Hennig, Maximiliane Jansky, Michelle Kaufmann, Markus Krause, Sabine Krauss, Thomas Lehmann, Helmut L’hoest, Srikanth Maddela, Ursula Marschall, Martial Mboulla, Winfried Meißner, Heiner Melching, Cornelia Nageler, Friedemann Nauck, Sara Parhizkari, Judith Rothaug, Joachim Saam, Werner Schneider, Sven Schulz, Kathleen Stichling, Horst C. Vollmar, Julia von Hayek, Ulrich Wedding

**Affiliations:** 1 Institute of General Practice and Family Medicine, Jena University Hospital, Bachstraße 18, 07743 Jena, Germany; 2 Center for Interdisciplinary Health Research, University of Augsburg, Universitätsstraße 2, 86159 Augsburg, Germany; 3 Clinic for Palliative Medicine, University Medical Center Göttingen, Von Siebold-Str. 3, 37075 Göttingen, Germany; 4 Center for Clinical Studies, Jena University Hospital, Salvador-Allende-Platz 27, 07747 Jena, Germany; 5 Department of Medicine and Health Services Research, BARMER Statutory Health Insurance Fund, Lichtscheider Straße 89, 42285 Wuppertal, Germany; 6 Institute of General Practice and Family Medicine, Faculty of Medicine, Ruhr University Bochum, Universitätsstraße 150, 44801 Bochum, Germany; 7 Department of Palliative Care, Jena University Hospital, Am Klinikum 1, 07747 Jena, Germany

**Keywords:** Palliative care, End-of-life care, Specialized outpatient palliative care, Ambulatory care, Patient reported outcomes, General practitioner, Quality of care, Mixed methods, Claims data, Health service research

## Abstract

**Background:**

Since 2007, the German statutory health insurance covers Specialized Outpatient Palliative Care (SAPV). SAPV offers team-based home care for patients with advanced and progressive disease, complex symptoms and life expectancy limited to days, weeks or months. The introduction of SAPV is ruled by a directive (SAPV directive). Within this regulation, SAPV delivery models can and do differ regarding team structures, financing models, cooperation with other care professionals and processes of care. The research project SAVOIR is funded by G-BA’s German Innovations Fund to evaluate the implementation of the SAPV directive.

**Methods:**

The processes, content and quality of SAPV will be evaluated from the perspectives of patients, SAPV teams, general practitioners and other care givers and payers. The influence of different contracts, team and network structures and regional and geographic settings on processes and results including patient-reported outcomes will be analyzed in five subprojects: [1] structural characteristics of SAPV and their impact on patient care, [2] quality of care from the perspective of patients, [3] quality of care from the perspective of SAPV teams, hospices, ambulatory nursing services, nursing homes and other care givers, content and extent of care from [4] the perspective of General Practitioners and [5] from the perspective of payers.

The evaluation will be based on different types of data: team and organizational structures, treatment data based on routine documentation with electronic medical record systems, prospective assessment of patient-reported outcomes in a sample of SAPV teams, qualitative interviews with other stakeholders like nursing and hospice services, a survey in general practitioners and a retrospective analysis of claims data of all SAPV patients, covered by the health insurance fund BARMER in 2016.

**Discussion:**

Data analysis will allow identification of variables, associated with quality of SAPV. Based on these findings, the SAVOIR study group will develop recommendations for the Federal Joint Committee for a revision of the SAPV directive.

**Trial registration:**

German Clinical Trials Register (DRKS): DRKS00013949 (retrospectively registered, 14.03.2018), DRKS00014726 (14.05.2018), DRKS00014730 (30.05.2018). Subproject 3 is an interview study with professional caregivers and therefore not registered in DRKS as a clinical study.

## Background

Corresponding to the Federal Statistical Office about 1% of the population in Germany dies within a year [1] and studies as well as the German Association for Palliative Medicine (DGP) estimate that about 90% of these require some level of palliative care [2–5]. To facilitate understanding of palliative care provision in the German health care system in the international community, we use the terminology defined by the European Association for Palliative Care (EAPC) in the “White Paper on standards and norms for hospice and palliative care in Europe” [6, 7]. According to the EAPC, palliative care can be made available on three different levels: palliative care approach, general palliative care and specialist palliative care – whilst palliative care approach and general palliative are often dealt as a joint category when focusing on the contrast to specialist palliative care, as we will do during this study protocol. While most patients with a life-limiting disease may only require general palliative care as provided by primary care professionals or specialists, a substantial amount of patients suffer from a high burden of symptoms and therefore require specialist palliative care. In case of outpatient care in Germany, general palliative care can be provided as General Outpatient Palliative Care (AAPV) and patients with a need for specialist palliative care can be referred to Specialized Outpatient Palliative Care (SAPV). SAPV is regularly provided by multi-professional home palliative care teams, comprising at least physicians and nurses, delivering specialized palliative care at the homes of patients with complex needs and suffering from incurable, progressive and life-limiting diseases.

Since the German Act to Strengthen Competition in Statutory Health Insurance (GKV-WSG) was introduced in 2007, SAPV has been fully covered by the Statutory Health Insurance [8] and can be prescribed by outpatient physicians as general practitioners (GPs),or by hospital physicians. Eligibility criteria as well as type and extent of SAPV services are determined by the SAPV directive of the Federal Joint Committee (G-BA, the highest decision-making body of the joint self-government of physicians, dentists, hospitals and health insurance funds in Germany) [9]. The SAPV directive defines objectives of SAPV (§1), eligibility requirements (§2, §3), particularly complex care (§4), content and scope of SAPV (§5), collaboration with other care providers (§6), prescription of SAPV (§7) and the assessment of entitlement for benefits by health insurance funds (§8). This directive has been worded in general terms to enable health insurance funds and care providers to make individual agreements (selective contracting), based on regional specifications and already existing structures. The resulting regulations are highly variable regarding care provision, cooperation and quality assurance as well as financial reimbursement and has led to different models [10]. This is reflected in over 290 different SAPV contracts nationwide [11], so that team structures, cooperation with other care givers, patient inclusion and exclusion criteria, processes of care, treatment intensity and financing models may differ substantially between the SAPV teams.

In 2016 about 55.000 patients (6% of the deceased in Germany) had received SAPV [11] before their death. This proportion is well below the estimated 10% of dying patients with a need for specialist palliative care at home [2]. However, recent analyses in Germany focused mainly on the description of (regional) SAPV-structures and utilization [12–16] as, for instance, the study “Faktencheck Gesundheit” investigated regional disparities of SAPV prescriptions [17]. Until now, no nationwide assessment of SAPV from different perspectives has been conducted [18]. In our view, there is a special need for an evaluation of the SAPV directive taking into account:

1. The heterogeneity of organizational structures and processes and their effects on patient care: Heterogeneity is particularly represented by different forms of ownership, team structure, integration of different professions, reimbursement systems and regional factors like urban or rural settings [14]. This also leads to different levels of involvement of the SAPV teams (consultation, coordination of care, partial or full provision of care). The potential consequences of these different contractual elements on patients´ care are mostly unknown.
2. As palliative care is a multi-professional, patient-centered care model, different perspectives of affected persons and involved professionals should be considered [19].
  - Although the individual needs of patients and their relatives are key aspects of the SAPV directive, there are no formal requirements for a corresponding quality assurance. Available research does not focus on the patient perspective at all [17] or only does so in specific scenarios [14, 20]. Assessing quality of care without consideration of the patients perspective will remain incomplete [21]. With the help of the questionnaire QUAPS (Quality Assurance in Outpatient Palliative Care) we will be able to investigate quality of SAPV from patients’ perspective [22].
  - Since SAPV is acting in a complex social environment of patients, relatives and health care professionals, the evaluation of overall quality of palliative care requires an adapted and differentiated analysis considering cooperation and perspective of these stakeholders. Accordingly, SAPV is a process of interactions of these participants, located in the individual patient’s environment. Therefore it is necessary to describe the local cooperation of health care professionals.
  - General practitioners (GP) are substantially involved in the end of life care of their patients [23]. They play an important role in AAPV, can introduce SAPV to patients’ care by medical prescription and often remain subsequently involved [24]. This key role strengthens the need of assessing the impact of GPs on the quality of SAPV in connection to patients, other health professionals and the SAPV directive.
3. The interface between general and specialist outpatient palliative care appears to be under-explored [11, 25]. Furthermore, available claims data studies focused on a small selection of specific aspects of palliative care [26]. Investigation of utilization and costs of different types of palliative care using claims data is a useful addition. On that basis it might be possible to find hints for over-, under- and missupply of end-of-life-care considering health economic perspectives.
4. Against the background of the requirements of the national ethics council [27], it is an important goal to highlight the patients’ welfare as the leading principle in medical care. Taking this into account, it is necessary to focus on potential shortage of outpatient palliative care in specific patient groups, for example patients with non-malignant disorders.

So far, a systematic and nationwide data assessment in SAPV is lacking. In order to support the further development of SAPV and the guiding SAPV directive, it is necessary to assess the process, extent and quality of SAPV and their determining factors. For this purpose, SAVOIR (Evaluation of the SAPV directive, Outcomes, Interactions and regional variations) was funded by the German Innovations Fund (launched by the Federal Joint Committee) to evaluate the execution of the SAPV directive and to present suggestions for a revision of the SAPV directive.

The project is performed by the Department of Palliative Care (coordinator), Institute of General Practice and Family Medicine (both Jena University Hospital), Clinic for Palliative Medicine (University Medical Center Göttingen), Center for Interdisciplinary Health Research (ZIG, University of Augsburg), German federal association for SAPV (BAG-SAPV), German Association for Palliative Medicine (DGP) and the health insurance fund BARMER.

## Methods/design

### Aim of the study

The project will study care provision in SAPV from the perspective of patients, SAPV teams, other care givers, GPs and payers. The influence of different contracts, team and network structures, regional and geographic settings on processes and results including patient-reported outcomes will be analyzed. This will be carried out in five subprojects:

**Subproject 1:** The aim is to investigate the structural characteristics of SAPV (determined by contracts and organizational team structures) and assess if and how these aspects influence patient care.

**Subproject 2:** The aim is to investigate the quality of care from the perspective of patients.

**Subproject 3:** The aim is to estimate the quality of care from the perspective of SAPV teams and external network partners such as hospices, ambulatory nursing services, nursing homes and other care givers.

**Subproject 4:** The aim is to investigate the type and extent of GPs palliative care with and without SAPV and the quality of palliative care from their perspective.

**Subproject 5:** The aim is to describe and distinct different types of palliative care, the patients receiving these types of care, their health care utilization and quality of care in its regional variations by means of health insurance fund claims data.

Primarily, mixed-methods data analysis will allow the identification of variables that influence care provision and may be associated with quality of SAPV. Based on these findings, the SAVOIR study group will provide recommendations for the revision of the SAPV directive for the Federal Joint Committee.

### Design and setting

The project will be based on different data sources: subproject 1a) registry data on team and organizational structures from the German Guide for hospice and palliative care services as well as model contracts in all German regions and 1b) clinical routine patient data from electronic medical record (EMR) of a sample of SAPV teams, subproject 2) patient-reported outcomes in a sample of about 50 SAPV teams, each collecting data from 25 patients, subproject 3) qualitative interviews with members of SAPV teams and linked providers involved in SAPV, like nursing and hospice services, subproject 4) survey data from GPs and subproject 5) health insurance fund claims data from patients who have died in 2016 and were covered by the health insurance fund BARMER.

This study is conducted in Germany from July 2017 to June 2019 and includes a transdisciplinary and multi-method design. The overall project is characterized by a cross-project use of data and a combination of quantitative and qualitative research designs. The focus of the quantitative research is obtaining a representative cross-section of the German SAPV, for instance by mapping the 17 Regional Associations of Statutory Health Insurance Physicians (KV). These regions are equivalent to the 16 Federal German States, but North Rhine-Westphalia is divided into North Rhine and Westphalia-Lippe.

From a sample of about four SAPV teams per KV, aggregated data of approximately 70 patients per SAPV team are associated to structural characteristics of the selected teams (subproject 1). Following this, a patient survey of a sub-group of about 25 patients per SAPV team will be conducted, focusing on the quality of care from the perspective of patients and on linking these findings to individual demographic and clinical data (subproject 2). A postal survey of about 1200 GPs using a standardized, quantitative study design in eight KV domains is conducted to explore their involvement in and needs regarding SAPV (subproject 4). An analysis of palliative care based on claims data [17, 28], provided by the health insurance fund BARMER, is performed to represent the utilization of the different types of in- and outpatient palliative care services in Germany (subproject 5). Data which cannot be obtained via quantitative research designs – such as interpretations and evaluation categories of quality of care by SAPV teams and their network of care givers and service providers – will be explored by a qualitative design. This is carried out as an on-site field study involving comparative case studies. Ten SAPV teams, each of which represents a minimal or maximal type within a set of contrasting structural characteristics, will be explored by means of field trips of several days duration (subproject 3). Wherever possible, synergies between the SAVOIR subprojects will be used. This involves the exchange of data (e.g. regarding the empirically grounded selection criteria for SAPV teams) and cooperation regarding content and concept of the subprojects.

### Data management

The data management and all required steps of data preparation (for example check for plausibility and integrity) will be located at the Center for Clinical Trials at University Hospital Jena. All participant data will be pseudonymized. In addition, data collected from the subprojects will be managed at the corresponding institution: in subproject 4, data from the survey will be anonymously evaluated. The claims data analysis in subproject 5 will be based on the recommendations of the “Good Practice Secondary Data Analysis” [29]. The data management of subproject 3 will be located at the Center for Interdisciplinary Health Research (ZIG) at the University of Augsburg.

### Ethics

Votes from the local ethics committees of the respective institutions were obtained from Jena University Hospital (No. 5312–10/17, 5316–10/17, 5317–10/17), University Medical Center Göttingen (No. 31/8/17) and University of Augsburg (06.09.2018).

### Sample and procedure

#### Subproject 1

Structural characteristics of SAPV and their impact on quality of care.

##### Hypotheses

Differences in provision of care are associated with different team structures and regulatory foundations.

##### Method and Design

The study encompasses three steps. Step 1a: Assesses SAPV structures nationwide (in cooperation with the DGP). Step 1b: Assesses regulatory structures, specifically the contracts between health insurance funds and SAPV teams (in cooperation with the BAG-SAPV). Step 2: SAPV teams provide patient data prospectively for 6 months. The patient data will be analyzed regarding regional and structural differences.

##### Study population

Step 1a/1b: Comprehensive assessment of SAPV teams and contracts. Step 2: Recruitment of 4 teams per region (*n* = 17). Assessment of 70 patients per team in six months, resulting in 5.950 patient data sets.

##### Data collection

Step 1a: In 2017, all institutions providing SAPV in Germany were asked to submit data to the guide for hospice and palliative care services (Wegweiser “Hospiz- und Palliativversorgung”), an online data base provided by the DGP. The guide is based on voluntary information given by institutions and contains data of 270 SAPV providers. As there is no existing comprehensive list of SAPV providers in Germany, the guide contains the largest existing list of SAPV teams. Step 1b: The contracts will be analyzed regarding their differences in regulation. In order to validate information and assess different practices, interviews with local SAPV providers or stakeholders, who are involved in policy making, are conducted. Step 2: Coded patient data is extracted through two widely used electronic medical records (EMR) software solutions in Germany: PalliDoc (StatConsult) and ISPC (Smart-Q). Data assessment is based on the consented data set for a nationwide hospice and palliative care registry. Few additional items will be assessed (number of visits, number of hospital additions, socioeconomic status). SAPV teams ask all patients admitted to SAPV during six month for permission for data extraction.

##### Data analyses

Step 1a/1b: Structural and contract variables will be analyzed descriptively. Step 2: Hierarchical multilevel models will be used to analyze associations between structural predictors and the patient and care parameters.

#### Subproject 2

Quality assurance in SAPV – a quantitative collection of data from patients´ perspective.

##### Hypotheses

Patients´ perspective on quality of care in SAPV is correlated to structures and processes of SAPV teams.

##### Method and Design

With a questionnaire, called QUAPS [22], developed to assess quality of care with SAPV, patients report their perspective on quality of symptom control, processes of care and results. In addition, IPOS (Integrated Palliative care Outcome Scale) is assessed at the time of inclusion (IPOS baseline) and about 3–14 days after inclusion (IPOS follow-up).

##### Study population

Data from 25 consecutive patients from 50 SAPV teams covering the 17 KV regions are collected over a maximal period of 6 months, resulting in data from 1250 patients.

##### Data collection

Data of all patients cared for by the SAPV team within the period are collected with subproject 1 via export from the EMR system (PalliDoc or ISPC). The IPOS and QUAPS questionnaires are collected by the local SAPV team and sent in a closed envelope to the study center.

##### Data analyses

Data analysis is descriptive. IPOS baseline data are compared to IPOS follow-up data. QUAPS data are compared between SAPV teams. In a further step, data of subproject 2 are analyzed for their association with data on structures and processes of SAPV teams, collected by subproject 1.

#### Subproject 3

Beneficial and inhibiting factors for a successful performance of SAPV teams from the perspective of SAPV team members and network partners.

##### Hypotheses

The self-perception, working strategies, team cultures and organizational structures of SAPV teams are influenced by patient-, payment-, organization-, region- and structure-related factors.

##### Method and Design

We develop and carry out problem-centered interviews within the framework of an ethnographic qualitative study design by means of on-site field studies based on grounded theory. The aim of grounded theory research is not only to “uncover relevant conditions but also to determine how the actors under investigation actively respond to those conditions and to the consequences of their actions” [30]. Basic elements of a research approach according to the grounded theory are the following: Data analysis begins with the first bits of data available, further collection of data is based on the ongoing process of data analysis all along the duration of a research project. Any outcome of the data analysis such as concepts and categories are constantly contrasted and compared, e.g. similar situations discovered in various interviews are compared. This is also an important means to evaluate the findings. Thus, concepts discovered at the beginning of the research process are likely to have completely changed till the end of the research.

##### Study population

The study population is centered on 10 SAPV teams and their environment, i.e. their external network partners as well as other local care providers - if possible even those who deliberately chose not to cooperate with the SAPV team. The selection is determined by various categories such as size of team, economic prosperity of region, population density, organizational contexts, etc. The aim is to gather information from a broad spectrum of teams and to contrast certain categories as much as possible, for example a contrast between teams in rural vs. teams in urban areas or completely autonomous teams vs. teams embedded in a wider organizational context like hospitals or hospices.

##### Data collection

The data is being collected mainly by means of qualitative, problem-centered interviews. Interviews are being conducted on-site with SAPV team members such as team-managers, physicians, palliative care nurses, counselors and external network partners like GPs, retirement homes, palliative care units in hospitals, pharmacies and medical stores. Topics of the interview guidelines include daily working habits, characteristics of patients and team dynamics as well as future and structural effects of SAPV in the local area. The research-team also uses the technique of field notes to provide a wider context around the interviews.

##### Data analysis

We are conducting a qualitative analysis within the grounded theory framework. This involves inductive and deductive analysis of interview data.

#### Subproject 4

General Practitioners‘care for palliative patients within and outside SAPV (GPs‘perspective).

*Hypotheses:* The self-perception and tasks of GPs in palliative care in general and the prescription of SAPV by GPs in particular are determined by patient-, physician-, qualification-, payment-related and other environmental (i.e. palliative infrastructure) factors.

##### Method and Design

Based on a literature search for criteria to measure the role of the GP in palliative care, the following steps are part of the project: Step 1: Definition and evaluation of corresponding questions for the qualitative analysis of interviews with GPs in cooperation with subproject 3 and in preparation for the subsequent quantitative analysis. Step 2: Quantitative analysis of a GP survey. Step 3: Synthesis of the results of the qualitative and quantitative analysis as well as the claims data analysis (subproject 5) with the aim to derive criteria for a systematic description of the GP’s involvement in AAPV and SAPV. The development of the questionnaire (step 2) is based on a literature search and semi-structured interviews with GPs and palliative care specialists. Pretests are performed before finalizing the questionnaire.

##### Study population

Random sample of 6000 GPs in the federal states of Bavaria, Berlin, Hesse, Lower Saxony, Saxony-Anhalt, Schleswig-Holstein, Thuringia and Westphalia-Lippe as a part of North Rhine-Westphalia. The selection is based on the structure of regional SAPV contracts as well as the number of inhabitants and size to represent the heterogeneity of SAPV in Germany.

##### Data collection

The GP address data are collected via free-access internet portals or obtained from the Regional Association of Statutory Health Insurance Physicians. The postal questionnaire is addressed to 750 GPs in each survey area. Survey data will be transcribed into a database electronically. Within a standardized, quantitative study design, a postal survey with 750 GPs from eight regions each is performed in 2018. The regions were selected in consideration of the different structural characteristics of SAPV in Germany, evaluated in subproject 1, to represent differences in the regions. The expected response rate is 20% (*n* = 1200).

##### Data analyses

We will conduct a descriptive analysis of quantitative variables and will also perform univariate and multivariate regression analyses on the association between independent and dependent variables.

#### Subproject 5

SAPV in comparison to other types of palliative care: patient characteristics and resource utilization (payer’s perspective).

*Hypotheses:* The type, extent and quality of palliative care a patient receives during the last months before death differs according to patient characteristics as well as regional structures of palliative care provision.

##### Method and Design

We perform a retrospective cohort study in a cross-sectional design with a subgroup analysis. By means of pseudonymized master data, people insured with the health insurance fund BARMER who died in 2016 (study population) are identified. It will be determined if patients received palliative care and if yes, what level of palliative care with regard to general or special palliative care, in- or outpatient palliative care, or combinations thereof, they received within the last six months before their death. On this basis, we will assign the patients to the study cohorts.

##### Study population

About 95,000 adults throughout Germany, insured, with the health insurance fund BARMER and time of death in the year 2016.

##### Data collection

Pseudonymous individual claims data (according to §§ 284, 295, 300, 301, 302 of the Fifth Book of the Social Code, SGB V) are made available via the Scientific Data Warehouse of the health insurance fund BARMER. Additional variables and value lists, which are considered relevant for the planned investigation (i.e. specific billing codes for AAPV and SAPV as well as hospices care data), were supplemented. Relevant periods of observation of received health insurance fund services include the last six and three months before death, the time period between months 12 and seven before death is defined as pre-observation period. The assignment of patients to cohorts and subgroups is executed by means of defined outpatient billing codes or hospital-based operation and procedure codes (OPS). We use preexisting indicators and – under consideration of the limitations of claims data - develop and quantify new and eligible indicators to describe distinct types of palliative care, their utilization and their quality of care. The study population will be characterized by socio-economic, administrative diagnoses and other care-specific attributes. In addition, we will calculate measures aimed at describing the quality of palliative care based on variables available in claims data.

##### Data analyses

Outcome measures and patient characteristics will be described statistically for each cohort and region. For the comparison of outcome measures between cohorts and regions we intend to use multivariate regression analyses.

## Discussion

Recent analyses in Germany focused mainly on the description of (regional) SAPV structures and utilization [12–15, 17]. A nationwide assessment of SAPV from different and relevant perspectives has not yet been conducted. To provide this, SAVOIR addresses: [1] the structural characteristics of SAPV and their impact on patient care, [2] the quality of care from the perspective of patients, [3] the quality of care from the perspective of SAPV teams, hospices, ambulatory nursing services, nursing homes and other care givers, [4] the content and extent of care from the perspective of GPs and [5] the health insurance fund as a payer. The comprehensive, nationwide data assessment in the SAVOIR study uses different perspectives and data sources as well as both quantitative and qualitative methods. This allows us to identify the current conditions and special needs in palliative care from patients, relatives, care providers and payers in Germany. Subsequently we will compare different care models and provide an overview of SAPV provision in Germany and moreover we will provide an in-depth analysis of differences - even if we will not be able to take the entirety of differences into account. Based on these findings, factors related with high quality of care in SAPV will be identified.

Although SAVOIR uses multi-method and transdisciplinary approach with unique advantages, clinical research in patients with an advanced and life-limiting disease remains challenging, i.e. with regard to suitable research methods as well as the participation of healthcare professionals and the recruitment of patients [31–34].

According to the different research strategies in each subproject, we are conscious of the following limitations:

**Subproject 1:** In step 1a, teams may not provide their real personnel structures, but rather a socially desirable version. The interviews were conducted via phone, which may increase this effect. The regulatory contracts may not cover the “real care provision”, as there may be regional differences that were not captured in the interviews and that we are unaware of. Some regions have model contracts, but specific characteristics like financial reimbursement may still vary between teams. To assess patient care, we use clinical routine data. While our prospective assessment enables us to give the teams information about the extracted data in advance and thus provide a better data quality, clinical routine data still is difficult to interpret. The variables used may be differently understood by those who document them and thus result in various interpretations within and between collecting SAPV teams.

**Subproject 2:** Questioning palliative patients and their relatives can only be pursued under several limitations: Patients are often in a poor general condition, participation in research is an additional burden for them and palliative care teams are often not experienced in clinical research and are reluctant to address participations. A specific challenge is given by the fact that life expectancy in SAPV patients is often limited to days or few weeks and treatment ends in most cases with the death of the patient. Therefore, in contrast to most other types of care, it is not possible to approach patients at the end of SAPV treatment. On the other hand, patients have to have experienced a certain time of SAPV care before they can report on its quality. Therefore, there is only a small time window to obtain patient-reported information on quality of SAPV care [35]. This may reduce the sample size and those participating may be a selected group of relatively “healthy” patients. However, pilot testing has revealed that the chosen approach will result in a sufficient number of data sets. Therefore we conduct a multi-method design, collecting treatment data of all patients agreeing to participate in subproject 1 over the time of recruitment for subproject 2.

**Subproject 3:** The field survey may lead to self-selection of participating SAPV teams: We expect that SAPV teams interested in research and improvement of palliative care will be more likely to participate. A measure to prevent self-selection of SAPV teams is the purposeful selection of participants via a set of various criteria such as the size of the team, economic prosperity of region, population density, organizational contexts, etc. Furthermore SAPV teams themselves select external network partners for the interviews. We expect that only external network partners are chosen for participation who are highly motivated, interested in palliative care and have positive experiences in working together with SAPV teams. Especially GPs with a more reserved attitude towards SAPV are unlikely to participate. This aspect is methodologically reflected during the data analysis.

**Subproject 4:** In the GP survey we have to deal with the fact that GPs more interested, qualified and/or experienced in end-of-life care might be more willing to participate in the survey than others. Therefore, we request information on the extent, experience and qualification in terms of palliative care from the GPs to be able to determine the impact of these factors on the results.

**Subproject 5:** Though the BARMER made great efforts to collect all relevant data on SAPV and to transfer it into the Scientific Data Warehouse, the claims data base on SAPV will not be fully complete by the end of the project and may not cover all existing health services within SAPV in Germany. The data do not contain full information on the qualification levels of care providers necessary to finer differentiate between levels of palliative care such as palliative care approach and general patient care. Apart from many advantages in analyzing claims data, their information value for the measurement of quality of care is restricted due to the lack of clinical and patient reported data.

To sum up, SAVOIR – consisting of five subprojects – will provide important information on the complex and regionally varying structures, processes, extent and quality of SAPV in Germany and the factors which determine the quality of SAPV from the different perspectives of stake-holders involved. We aim at deriving recommendations to further develop the SAPV directive. The results will add valuable knowledge on starting points to improve outpatient palliative care, mainly in Germany, that might be applicable for other countries, too.

## Abbreviations

AAPV: General Outpatient Palliative Care
BAG-SAPV: German working group for SAPV
BQKPMV: Particular Qualified, Coordinating Palliative Medical Care
DGP: German Association for Palliative Medicine
EAPC: European Association for Palliative Care
EMR: Electronic Medical Record
G-BA: Federal Joint Committee, the highest decision-making body of the joint self-government of physicians, dentists, hospitals and health insurance funds in Germany
GKV-WSG: German Act to Strengthen Competition in Statutory Health Insurance
GPS: Good Practice Secondary Data Analysis
IPOS: Integrated Palliative care Outcome Scale
KV: (regional) Association of Statutory Health Insurance Physicians
OPS: Operation and Procedure Codes
QUAPS: Quality Assurance in Outpatient Palliative Care
SAPV: Specialized Outpatient Palliative Care
SGB: Social Code Book

## References

[1] Statistisches Bundesamt. Sterbefälle 2016. Statistisches Bundesamt (Destatis); 2018 [27.06.2018]; Available from: https://www.destatis.de/DE/ZahlenFakten/GesellschaftStaat/Bevoelkerung/Sterbefaelle/Sterbefaelle.html#Tabellen. Accessed 24 Jan 2019.

[2] Deutsche Gesellschaft für Palliativmedizin. Stellungnahme der Deutschen Gesellschaft für Palliativmedizin: SAPV: Die spezialisierte Ambulante Palliativversorgung ist kein Wettbewerbsfeld! : Deutsche Gesellschaft für Palliativmedizin; 2017.

[3] McNamara B, Rosenwax LK, Holman CDAJ (2006). A method for defining and estimating the palliative care population. J Pain Symptom Manag.

[4] Murtagh FE, Bausewein C, Verne J, Groeneveld EI, Kaloki YE, Higginson IJ (2014). How many people need palliative care? A study developing and comparing methods for population-based estimates. Palliat Med.

[5] Scholten N, Günther AL, Pfaff H, Karbach U (2016). The size of the population potentially in need of palliative care in Germany - an estimation based on death registration data. BMC Palliat Care.

[6] Radbruch L, Payne S (2009). EAPC Board of Directors. White paper on standards and norms for hospice and palliative care in Europe: part 1. Recommendations from the European Association for Palliative Care. Eur J Palliat Care.

[7] Radbruch L, Payne S (2010). EAPC Board of Directors. White paper on standards and norms for hospice and palliative care in Europe: part 2. Recommendations from the European Association for Palliative Care. Eur J Palliat Care.

[8] Busse R, Blumel M, Knieps F, Barnighausen T (2017). Statutory health insurance in Germany: a health system shaped by 135 years of solidarity, self-governance, and competition. Lancet.

[9] Gemeinsamer Bundesausschuss. Richtlinie des Gemeinsamen Bundesausschusses zur Verordnung von spezialisierter ambulanter Palliativversorgung. 2010.

[10] Jansky M, Lindena G, Nauck F (2011). Stand der spezialisierten ambulanten Palliativversorgung (SAPV) in Deutschland – Verträge und Erfahrungen. Zeitschrift für Palliativmedizin..

[11] Gemeinsamer Bundesausschuss (2016). Bericht an das Bundesministerium für Gesundheit über die Umsetzung der SAPV-Richtlinie für das Jahr 2016.

[12] Schneider W (2012). Mehr als Symptomkontrolle Wirksamkeit in der SAPV. Evidenz und Versorgung in der Palliativmedizin: medizinische, psychosoziale und spirituelle Aspekte.

[13] Melching H (2015). Palliativversorgung: Strukturen und regionale Unterschiede in der Hospiz- und Palliativversorgung.

[14] Schneider W, Eichner E (2014). Struktur- und Prozesseffekte der SAPV in Bayern - Evaluation / Qualitätssicherung und (Aus-)Wirkungen der SAPV auf die AAPV (unter besonderer Berücksichtigung des ländlichen Raums).

[15] Bretschneider K, Kasprick L, Luderer C (2012). “Elisabeth Mobil mbH” – die spezialisierte ambulante Palliativversorgung im Raum Halle (Saale) – eine wissenschaftliche Auswertung. Zeitschrift für Palliativmedizin..

[16] Grabenhorst U. Specialized ambulatory palliative care: (SAPV) 5-year results of a multi-professional care model by HomeCare linker Niederrhein gGmbH (HC) in the lower Rhine region. Ann Oncologia. 2017;28(Suppl 5).

[17] Radbruch L, Andersohn F, Walker J. Palliativversorgung: Überversorgung kurativ – Unterversorgung palliativ? Analyse ausgewählter Behandlungen am Lebensende. Bertelsmann Stiftung, 2015.

[18] Nauck F, Jansky M (2018). Palliative home care teams in Germany. Spezialisierte Ambulante Palliativ-Versorgung. Dtsch Med Wochenschr.

[19] Stadelbacher S, Eichner E, Schneider W (2015). Praxis der spezialisierten ambulanten Palliativversorgung. Klinische Sozialarbeit.

[20] Schneider N. Anforderungen an eine patientenorientierte ambulante Palliativversorgung: Bertelsmann Stiftung; 2015.

[21] Koch A, Gaser E, Gretzinger S, Hempel CM, Wedding U, Meißner W. Qualitätssicherung in der Ambulanten PalliativverSorgung (QUAPS). Zeitschrift für Palliativmedizin. 2012;13(05).

[22] Hammer U, Berghaus D, Wedding U, Meißner W. QUAPS II – Qualitätssicherung in der Spezialisierten Ambulanten PalliativVersorgung (SAPV) – Entwicklung und Testung eines Tools – Erste Ergebnisse von QUAPS II. Zeitschrift für Palliativmedizin. 2016;17(05).

[23] Bruns H, Terhorst A, Gesenhues S, Weltermann B. Ländliche Palliativmedizin – Prädiktoren für ‟dying at home”. Zeitschrift für Palliativmedizin 2014;15(03).

[24] Gágyor I, Lüthke A, Jansky M, Chenot JF (2013). [end of life care in general practice: results of an observational survey with general practitioners]. Hausärztliche Versorgung am Lebensende (HAVEL): Ergebnisse einer retrospektiven Beobachtungsstudie. Schmerz.

[25] Schneider N, Engeser P, Behmann M, Kuhne F, Wiese B. [Specialized outpatient palliative care. The expectations of general practitioners]. Spezialisierte ambulante Palliativversorgung. Die Erwartungen von Hausärzten. Schmerz 2011;25(2):166, 8–73. 10.1007/s00482-011-1037-0 21424329

[26] Gaertner J, Drabik A, Marschall U, Schlesiger G, Voltz R, Stock S (2013). Inpatient palliative care: a nationwide analysis. Health Policy.

[27] Richter-Kuhlmann E. Deutscher Ethikrat: Fokus: Patientenwohl. Dtsch Arztebl International. 2016;113(15):A-700-A-2.

[28] Brown CR, Hsu AT, Kendall C, Marshall D, Pereira J, Prentice M, Rice J, Seow HY, Smith GA, Ying I, Tanuseputro P (2018). How are physicians delivering palliative care? A population-based retrospective cohort study describing the mix of generalist and specialist palliative care models in the last year of life. Palliat Med.

[29] Swart E, Gothe H, Geyer S, Jaunzeme J, Maier B, Grobe TG, Ihle P (2015). [good practice of secondary data analysis (GPS): guidelines and recommendations]. Gute praxis Sekundardatenanalyse (GPS): Leitlinien und Empfehlungen. Gesundheitswesen.

[30] Corbin JM, Strauss A (1990). Grounded theory research: procedures, canons, and evaluative criteria. Qual Sociol.

[31] Grande GE, Todd CJ (2000). Why are trials in palliative care so difficult?. Palliat Med.

[32] Kars MC, van Thiel GJ, van der Graaf R, Moors M, de Graeff A, van Delden JJ (2016). A systematic review of reasons for gatekeeping in palliative care research. Palliat Med.

[33] Holm M, Alvariza A, Fürst C-J, Wengström Y, Årestedt K, Öhlen J, Goliath I (2017). Recruiting participants to a randomized controlled trial testing an intervention in palliative cancer care – the perspectives of health care professionals. Eur J Oncol Nurs.

[34] Campbell LC, Bailey C, Armour K, Perry R, Orlando R, Kinghorn P, Jones L, Coast J (2016). A team approach to recruitment in hospice research: engaging patients, close people and health professionals. Int J Palliat Nurs.

[35] Ammari ABH, Hendriksen C, Rydahl-Hansen S (2015). Recruitment and reasons for non-participation in a family-coping-orientated palliative home care trial (FamCope). J Psychosoc Oncol.

